# Analysis of germination characteristics and metabolome of *Medicago ruthenica* in response to saline-alkali stress

**DOI:** 10.3389/fpls.2025.1592555

**Published:** 2025-07-01

**Authors:** Xiaoli Wei, Jie Wang, Chengti Xu, Yuanyuan Zhao, Xiaojian Pu, Wei Wang, Guangxin Lu

**Affiliations:** ^1^ Academy of Animal Husbandry and Veterinary Science, Qinghai University, Xining, China; ^2^ Northwest Key Laboratory of Cultivated Land Conservation and Marginal Land Improvement, Ministry of Agriculture and Rural Affairs, Delingha, China

**Keywords:** *Medicago ruthenica*, adversity stress, saline-alkali land, seed germination, metabolomics

## Abstract

**Introduction:**

This study aimed to investigate the seed germination characteristics and metabolic response mechanisms of *Medicago ruthenica* under saline-alkali stress, with the goal of elucidating its physiological and molecular mechanisms of salt-alkali tolerance.

**Methods:**

It was systematically analyzed the germination characteristics of four *M. ruthenica* germplasm resources (YSZ, XHZ, Shoulu, and Longzhong 1) from different origins with various concentrations of individual salts (NaCl, Na_2_SO_4_, NaHCO_3_) and compound saline-alkali stress treatments. Additionally, the metabolite changes in the domesticated species under saline-alkali stress were examined using metabolomics technology.

**Results:**

The results indicated that low concentrations of NaCl stress did not significantly inhibit the germination of *M. ruthenica* seeds; rather, it promoted germination to some extent. In contrast, high concentrations of NaHCO_3_ and compound saline-alkali stress significantly inhibited both seed germination and seedling growth. The germination characteristics of *M. ruthenica* germplasm resources from different sources exhibit variability under saline-alkali stress. Domesticated species demonstrated strong tolerance to salt-alkali conditions. Metabolomic analyses indicated that saline-alkali stress significantly impacts key metabolic pathways, including amino acid metabolism, sugar metabolism, and lipid metabolism in *M. ruthenica* sprouts, with a notable increase in the accumulation of amino acids and their derivatives. Correlation analyses revealed that amino acids such as L-arginine, histidine, and glutamine are significantly positively correlated with germination rate and root length, suggesting that these amino acids play a crucial role in *M. ruthenica*’s response to saline-alkali stress.

**Discussion:**

This study provided a new theoretical foundation for understanding the salt-alkali tolerance mechanisms of *M. ruthenica* and serves as an important reference for breeding salt-alkali tolerant forage varieties and for the ecological restoration of saline-alkali land.

## 1 Introduction

Globally, over 1.4 billion hectares of land are affected by salinization, accounting for 10% of the total land area and distributed across more than 100 countries and regions. Secondary salinization is causing the area of soil salinization to continuously expand (Basak et al., 2022). China is one of the countries with a significant area of saline-alkali land, encompassing 7.6 million hectares, which represents approximately one-fifth of the nation’s total cultivated land area. Salinization leads to the accumulation of salt ions in the soil, severely affecting soil aeration and causing compaction and hardening. This results in a substantial reduction in water content, leading to physiological water deficiency in plants (Hailu and Mehari, 2021; Tarolli et al., 2024). Furthermore, the increase in soil salt ions inhibits the absorption of other nutrients by plants, resulting in poor plant development and consequently reduced yields or even plant death (Chele et al., 2021; Balasubramaniam et al., 2023). Saline-alkali stress not only affects seed germination and seedling growth but also negatively impacts physiological metabolism through mechanisms such as osmotic stress, ion toxicity, and oxidative stress (Zhang et al., 2025b).

In recent years, the cultivation of salt-tolerant crops has emerged as a significant technical approach for utilizing saline-alkali lands. This method requires minimal investment and yields rapid results, enabling the productive use of extensive areas of saline-alkali soil without necessitating engineering modifications, thereby achieving favorable economic benefits. Furthermore, the utilization of saline-alkali lands can also lead to improvements in their properties, significantly enhancing utilization efficiency. The salt tolerance of plants varies across different developmental stages (Nabi et al., 2025); notably, the seed germination stage is the most sensitive period to saline-alkali stress. The salt tolerance observed during this stage can serve as an indicator of a plant’s overall salt resistance (Sun et al., 2019; Ghosh et al., 2025). Research indicated that different plant species exhibit considerable variations in their responses to saline-alkali stress, with some adapting to these environments through mechanisms such as regulating osmotic substance accumulation, antioxidant enzyme activity, and ion balance (Zhang et al., 2025a).

*Medicago ruthenica* is an important leguminous forage species that is widely distributed in arid and semi-arid regions, such as northern China and the Mongolian Plateau. Due to its strong drought resistance, cold tolerance, and ability to withstand saline-alkali conditions, *M. ruthenica* holds significant ecological and economic value in grassland restoration, the improvement and utilization of saline-alkali soils, and the development of animal husbandry. Some studies have revealed the tolerance of *M. ruthenica* to abiotic stress and its population history currently (Wang et al., 2021; Yin et al., 2021; Wu et al., 2022b; Wang et al., 2025); however, most of these studies focused on the seedling stage of the species. The germination characteristics and metabolic regulatory mechanisms of its seeds under saline-alkali stress have not been sufficiently investigated.

In recent years, metabolomics technology has emerged as a powerful analytical tool widely applied in the field of plant stress physiology research. Through metabolomics analysis, the changes in plant metabolites under stress conditions can be comprehensively revealed, elucidating their adaptation mechanisms. Studies have indicated that the accumulation of osmotic adjustment substances, such as proline and betaine, is considered one of the essential strategies for plants to cope with saline-alkali stress (Meena et al., 2019; Karimi et al., 2025). Furthermore, saline-alkali stress can induce the production of numerous secondary metabolites in plants, including flavonoids and polyphenolic compounds, which play significant roles in antioxidation and signal transduction (Ikram et al., 2025). Additionally, saline-alkali stress can affect key metabolic pathways, such as amino acid metabolism, sugar metabolism, and lipid metabolism (Han et al., 2023). This study utilized four different sources of M. ruthenica species as materials and established various saline-alkali treatments with differing concentrations. We identified varieties with enhanced saline-alkali tolerance through screening germination indicators and used them for metabolomic analysis. This research was of significant importance in elucidating the saline-alkali tolerance mechanisms of *M. ruthenica* and in breeding saline-alkali tolerant varieties.

## 2 Materials and methods

### 2.1 Source of materials

A total of four germplasm resources of *M. ruthenica* (YSZ, XHZ, Shoulu, and Longzhong 1) were tested. Among these, the wild and domesticated *M. ruthenica* were provided by the Academy of Animal and Veterinary Sciences of Qinghai University. Both YSZ and XHZ belong to *Melilotoides archiducis-nicalai*, with YSZ collected in Huangyuan County, Qinghai Province, in 2008, and XHZ consisting of seeds collected in 2022 after five years of cultivation that began in 2018 in Ping’an District, Qinghai Province. The varieties Shoulu and *Medicago ruthenica* ‘Longzhong 1’ were provided by Gansu Agricultural University. On August 28, 2023, the variety “Shoulu” of *M. ruthenica*, bred by the team led by Yu Xiaojun from the Key Laboratory of Forage Germplasm Innovation and New Variety Breeding at the Ministry of Agriculture and Rural Affairs, Gansu Agricultural University, was approved by the National Herbage Variety Approval Committee. The variety “Longzhong 1” of *M. ruthenica* was approved by the Gansu Provincial Herbage Variety Approval Committee.

### 2.2 Experimental design

Distilled water was utilized to prepare three individual salts: NaCl, Na_2_SO_4_, and NaHCO_3_, along with a composite salt solution comprising these three salts in a 2:1:1 ratio, designated as treatments A, B, C, and D. Four germplasm resources—wild species, domesticated species, Shoulu, and Longzhong 1 were represented as a, b, c, and d, respectively. Each treatment group was established with three concentration gradients of 0.3%, 0.6%, and 1.2%, denoted as 1, 2, and 3, respectively. Distilled water served as the control group. Each treatment with 4 replicates, resulting in a total of 13 treatments, of which 1 were control treatments. **Table 1** below shows the pH values of each solution.

**Table 1:** The pH values of solutions with different saline-alkali types.

Different treatments	pH of solutions with different concentrations				
Salt-alkali solutions	0.3 %	0.6 %	1.2 %	pH	CK
NaCl	7.1	7.2	7.1	7.13 ± 0.06	7.0
Na_2_SO_4_	7.2	7.1	7.3	7.20 ± 0.10	7.0
NaHCO_3_	8.4	8.5	8.6	8.50 ± 0.10	7.0
NaCl:Na_2_SO_4_:NaHCO_3_ (2:1:1)	7.9	8.1	8.2	8.00 ± 0.15	7.0

Select uniform and plump seeds of *M. ruthenica* and use sandpaper to abrade the hard seed coats. Subsequently, disinfect the seeds by immersing them in a 75% ethanol solution for 30 sec, followed by rinsing with distilled water 3 to 5 times. After removing surface moisture with sterilized filter paper, evenly distribute the seeds in culture dishes lined with double-layer filter paper. The culture dishes should be cleaned with distilled water and subjected to disinfection in an oven at 120°C for over 3 h. Each culture dish contains 50 seeds. Add 5 mL of distilled water to the control culture dish and 5 mL of the corresponding mixed solution to the other stress culture dishes. Place the culture dishes in an artificial climate incubator set to a temperature of (25 ± 1)°C and a humidity level of (60 ± 5)%. During the experiment, weigh the dishes daily and add distilled water as necessary to maintain a constant weight.

According to the germination characteristics of *M. ruthenica*, approximately 1 g of germinated seedlings from different treatments of XHZ were selected. After rinsing with distilled water and drying, the seedlings were quick-frozen in liquid nitrogen for 15 min and subsequently stored at -80°C for metabolic group detection. Additionally, the apparent characteristics and microstructure were exclusively selected for the study of XHZ.

### 2.3 Determination indicators

During the experiment, the seed germination status was observed and recorded every 24 h, based on the emergence of radicle. The germination energy (GE) and germination rate (GR) were subsequently calculated. On the 8th day of the experiment, five *M. ruthenica* sprouts were randomly selected from each replicate, and the bud length (BL), root length (RL), fresh weight (FW), and dry weight (DW) were measured. Additionally, the dry-to-fresh weight ratio (T/R) was calculated. Samples were also collected on the 8th day for transmission electron microscope analysis.


GE=G4/Number of tested seeds×100%;

GR=G8/Number of tested seeds×100%;

In the formula, G4 represents the number of seed germinations within 4 days, and G8 represents the number of seed germinations within 8 days.

Transmission electron microscopy was conducted on 3 mm segments of radicle tip tissue from germinated seedlings, with each treatment replicated three times. Following sampling, the tissues were promptly fixed in a 5% glutaraldehyde solution and stored at 4°C in a refrigerator. The preparation of ultra-thin sections for transmission electron microscopy followed the methodology outlined by (Deyan and College., Z.Y, 2018). The cell ultrastructure was observed and photographed using a Hitachi H-7600 transmission electron microscope (Cuirong et al., 2015).

### 2.4 Wide-target metabolomic analysis

#### 2.4.1 Sample pretreatment and metabolite extraction

Based on the germination indicators, we selected XHZ on the eighth day of germination as the sample for subsequent metabolic analysis. These samples were immediately flash-frozen in liquid nitrogen and subsequently stored at -80°C for further analysis. All treatments were performed with three replicates. Metabolomics analysis was performed by Genedenovo Biotechnology Co., Ltd., Guangzhou, China, following a previously reported method (Chen et al., 2013). A 0.1g sample was weighed and pulverized in liquid nitrogen, followed by homogenization in 1 mL of icecold 80% methanol (v/v) containing 0.1% (v/v) formic acid. The mixture was incubated on ice for 30 min and then centrifuged at 14,000 × g for 30 min at 4°C. The supernatant was transferred to a new tube, and 0.1 mg/mL lidocaine (internal standard, Sigma-Aldrich, Shanghai, China) was added. The solution was diluted with LC–MS-grade water to adjust the methanol concentration to 53% (v/v), mixed thoroughly, and subjected to a second centrifugation at 14,000 × g for 30 min at 4°C. The samples underwent a second round of centrifugation under the same conditions (Want et al., 2013). The supernatant was collected, filtered through a 0.22-μm membrane and subjected to liquid chromatography tandem mass spectrometry (LC–MS/MS) analysis. All reagents were LC–MS grade and purchased from Thermo Fisher Scientific.

#### 2.4.2 LC-MS/MS analysis

Liquid chromatography-tandem mass spectrometry (LC-MS/MS) analysis was carried out using the ExionLC™ AD system (SCIEX) coupled with a QTRAP^®^ 6500+ mass spectrometer (SCIEX; AB Sciex, Framingham, MA, USA; distributed by Genedenovo, China). For chromatographic separation, the sample was injected onto an Xselect HSS T3 column (2.1 mm × 150 mm, 2.5 μm particle size; Waters, Milford, MA, USA). A 20-min linear gradient elution program was employed for both positive and negative ionization modes, with a constant flow rate of 0.4 mL/min. The mobile phase consisted of two components: Eluent A, which was 0.1% formic acid in ultrapure water, and Eluent B, which was 0.1% formic acid in acetonitrile (Thermo Fisher Scientific). The solvent gradient parameters were set as follows: 2% B for 2 min; a gradient from 2% to 100% B over 15 min; 100% B for 17 min; a decrease from 100% to 2% B in 0.1 min; and maintaining at 2% B for 20 min. The mass spectrometry conditions were adjusted to operate in both positive and negative polarity modes. In the positive ionization mode, the instrument settings were configured as follows: curtain gas pressure was set at 35 psi, collision gas was set to medium, ion spray voltage was 5500 V, source temperature was 550°C. Similarly, for the negative ionization mode, the curtain gas pressure remained at 35 psi, the collision gas was also set to medium, the ion spray voltage was adjusted to -4500 V, the source temperature was kept at 550°C (Zhang et al., 2024).

### 2.5 Statistical analysis

The experiment employs a completely randomized design, with values presented as means ± standard error (SE) derived from three replicates. Statistical analysis of germination indicators is conducted using Excel 2021. The *post-hoc* comparisons utilize Dunnett’s multiple comparison test, with significance set at P ≤ 0.05. Pearson’s bivariate correlation analysis is performed using the SPSS 23.0 software package, which facilitates the estimation of the correlation coefficient (r) between two comparative variables. Subsequent scatterplot matrix analysis aids in assessing the effects of multiple parameters under both saline-alkali stress and non-stress conditions. For the GC-MS data, peak area normalization in metabolite profiling is achieved by dividing the peak area of metabolites by the fresh mass of the sample and the peak area of libiol, resulting in the relative response ratio. A non-parametric ANOVA is conducted to effectively differentiate between non-saline-alkali stress treatment samples and saline-alkali stress treatment samples. Additionally, multivariate statistical analyses, including Principal Component Analysis (PCA) and Partial Least Squares Discriminant Analysis (PLS-DA), are executed using the metabolomics data analysis software MetaboAnalyst 3.0.

## 3 Results

### 3.1 The impact of saline-alkali stress on the seed germination of *M. ruthenica*

#### 3.1.1 Germination indicators

Germination energy refers to the percentage of seeds that have germinated out of the total number of seeds tested, specifically when the number of germinated seeds reaches its peak during the germination energy. In this study, the germination energy of *M. ruthenica* was assessed after four days of germination. A high seed germination energy indicates robust seed vitality, uniform germination, and consistent emergence. The germination energy of Shoulu under 0.6% NaCl stress is significantly higher than that of the other varieties (*P<0.05*). Furthermore, under the same concentration of different saline-alkali stresses, the germination energy of XHZ is also significantly greater than that of other varieties (*P<0.05*). Additionally, it is noteworthy that *M. ruthenica* exhibited no germination energy under 0.6% NaHCO_3_ stress and no germination energy under 1.2% concentration of various saline-alkali stresses. The germination energy of various varieties under different saline-alkali conditions decreases with the concentration increases. Notably, at low to medium concentrations, NaHCO3 exerts the most significant effect on the germination energy of the three types of M. ruthenica (**Table 2**).

**Table 2:** Germination energy of *Medicago ruthenica* under different concentration stresses.

Concentration	Salt-alkali solutions	Germplasm resources			
		a	b	c	d
0	CK	0.47 ± 0.01d	0.95 ± 0.01a	0.58 ± 0.01b	0.53 ± 0.01c
1	A	0.23 ± 0.01d	0.57 ± 0.01a	0.45 ± 0.01b	0.31 ± 0.01c
	B	0.33 ± 0.01b	0.65 ± 0.01a	0.23 ± 0.01c	0.20 ± 0.01d
	C	0.19 ± 0.01b	0.67 ± 0.02a	0.20 ± 0.01b	0.13 ± 0.01c
	D	0.19 ± 0.01d	0.52 ± 0.01a	0.39 ± 0.01b	0.26 ± 0.01c
2	A	0.03 ± 0.01c	0.07 ± 0.01b	0.09 ± 0.01a	0.02 ± 0.01c
	B	0.09 ± 0.01b	0.21 ± 0.01a	0.07 ± 0.01cd	0.06 ± 0.00d
	C	–	–	–	–
	D	0.03 ± 0.01c	0.18 ± 0.00a	0.05 ± 0.01b	0.03 ± 0.01c
3	A	–	–	–	–
	B	–	–	–	–
	C	–	–	–	–
	D	–	–	–	–

In the table, "-" indicates that the seed has not germinated, The same applies below. a:YSZ, b:XHZ, c:Shoulu, d:Longzhong 1.

The germination rate is defined as the percentage of germinated test seeds relative to the total number of test seeds, serving as a crucial indicator of seed quality. In this study, the germination rate was assessed over an 8-day period. A high germination rate coupled with strong germination potential suggests rapid and uniform emergence, resulting in robust seedlings. Conversely, a high germination rate with weak germination potential indicates uneven emergence, characterized by numerous weak seedlings. The germination rate of XHZ under varying concentrations and saline-alkali stresses is significantly higher than that of other varieties (*P<0.05*). Notably, none of the four *M. ruthenica* seeds germinated under 0.6% NaHCO_3_, 1.2% NaCl, 1.2% NaHCO_3_, or 1.2% compound saline-alkali stresses (**Table 3**). Among the different concentrations of *M. ruthenica*, the germination potential of three variants was equal to or exceeds that of the control group at low NaCl concentrations. However, as the concentration increases, the germination potential of *M. ruthenica* under various saline-alkali stresses exhibited a declining trend. Thus, at low NaCl concentrations, there was either no effect or a promotion of germination, whereas medium and high concentrations inhibit the germination of *M. ruthenica*. In contrast, other saline and alkali conditions, irrespective of concentration, consistently inhibit the germination of *M. ruthenica*. At low concentrations, the germination rate of different *M. ruthenica* was highest under NaCl stress. At medium concentrations, the germination rate of *M. ruthenica* was highest under mixed salt. At high concentrations, the germination rate of *M. ruthenica* was highest under Na_2_SO_4_ stress. It was noteworthy that among the germinated seeds, the XHZ exhibits a germination rate below 0.5 under the stress of 1.2% Na_2_SO_4_, while the germination rates under other saline-alkali stresses exceed 0.5.

**Table 3:** Germination rate of *Medicago ruthenica* under different concentration stresses.

Concentration	Salt-alkali solutions	Germplasm resources			
		a	b	c	d
0	CK	0.49 ± 0.01d	0.95 ± 0.01a	0.71 ± 0.01b	0.65 ± 0.01c
1	A	0.54 ± 0.01c	0.95 ± 0.01a	0.89 ± 0.01b	0.54 ± 0.01c
	B	0.48 ± 0.01c	0.93 ± 0.01a	0.63 ± 0.01b	0.45 ± 0.01d
	C	0.19 ± 0.01d	0.85 ± 0.02a	0.39 ± 0.01b	0.31 ± 0.01c
	D	0.48 ± 0.01c	0.94 ± 0.01a	0.70 ± 0.01b	0.51 ± 0.01c
2	A	0.35 ± 0.01b	0.65 ± 0.01a	0.35 ± 0.01b	0.24 ± 0.01c
	B	0.49 ± 0.01b	0.75 ± 0.01a	0.43 ± 0.01c	0.19 ± 0.01d
	C	–	–	–	–
	D	0.43 ± 0.01b	0.81 ± 0.01a	0.37 ± 0.01c	0.29 ± 0.01d
3	A	–	–	–	–
	B	0.07 ± 0.01c	0.43 ± 0.01a	0.05 ± 0.01c	0.13 ± 0.01b
	C	–	–	–	–
	D	–	–	–	–

a:YSZ, b:XHZ, c:Shoulu, d:Longzhong 1.

The plumule length of Longzhong 1 was significantly greater than that of YSZ (*P<0.05*) under the 0.3% Na_2_SO_4_ stress. Under 0.3% NaHCO_3_ stress, the plumule lengths of XHZ and Shoulu were significantly greater than those of YSZ and Longzhong 1 (*P<0.05*). Under 0.3% mixed saline-alkali stress, the plumule length of YSZ was significantly lower than that of the other varieties (*P<0.05*). Under 0.6% NaCl stress, the plumule length of Longzhong 1 was significantly lower than that of the other varieties (*P<0.05*). Under 0.6% mixed salt, the plumule length of Shoulu was significantly greater than that of Longzhong 1 (*P<0.05*). Under 1.2% Na_2_SO_4_ stress, the germ length of XHZ was significantly higher than that of YSZ and Shoulu (*P<0.05*). Regarding different concentrations of M. ruthenica, except for Na_2_SO_4_ stress, the germ length of M. ruthenica under other saline-alkali stresses decreased with increasing concentration. Under Na_2_SO_4_ stress, the germ length of the three types of M. ruthenica initially increased and then decreased with increasing concentration. Under the same concentration, NaHCO_3_ stress exerted the most pronounced inhibitory effect on the plumule length of various M. ruthenica species, followed closely by mixed conditions (**Table 4**).

**Table 4:** The germ length of *Medicago ruthenica* under different saline-alkali stresses.

Concentration	Salt-alkali solutions	Germplasm resources			
		a	b	c	d
0	CK	1.70 ± 0.20a	1.66 ± 0.11a	1.44 ± 0.05a	1.82 ± 0.12a
1	A	1.58 ± 0.20a	1.50 ± 0.08a	1.52 ± 0.04a	1.68 ± 0.16a
	B	1.18 ± 0.12b	1.38 ± 0.06ab	1.46 ± 0.10ab	1.52 ± 0.09a
	C	0.28 ± 0.08b	1.32 ± 0.13a	1.06 ± 0.11a	0.44 ± 0.07b
	D	0.84 ± 0.23b	1.36 ± 0.15a	1.50 ± 0.09a	1.42 ± 0.29a
2	A	1.26 ± 0.13a	1.50 ± 0.24a	1.28 ± 0.13a	0.70 ± 0.17b
	B	1.28 ± 0.13a	1.54 ± 0.16a	1.58 ± 0.09a	1.36 ± 0.09a
	C	–	–	–	–
	D	0.68 ± 0.15ab	0.70 ± 0.12ab	0.84 ± 0.12a	0.36 ± 0.05b
3	A	–	–	–	–
	B	0.40 ± 0.14b	0.76 ± 0.07a	0.27 ± 0.07b	0.52 ± 0.10ab
	C	–	–	–	–
	D	–	–	–	–

a:YSZ, b:XHZ, c:Shoulu, d:Longzhong 1.

As shown in **Table 5**, when germinated with distilled water, the radicle of Shoulu was the longest, significantly exceeding that of XHZ (*P<0.05*). Under 0.3% Na_2_SO_4_ stress, the radicle of Shoulu remains significantly longer than that of YSZ (*P<0.05*). In the presence of 0.3% NaHCO_3_ stress, the radicles of both Shoulu and Longzhong 1 are significantly longer than those of YSZ and XHZ (*P<0.05*). Similarly, under 0.3% mixed salt, the radicles of Shoulu and Longzhong 1 are significantly longer than that of YSZ (*P<0.05*). When subjected to 0.6% NaCl stress, the radicle of Shoulu is significantly longer than those of the other varieties (*P<0.05*). Under 0.6% Na_2_SO_4_ stress, the radicle of XHZ is significantly longer than that of YSZ (*P<0.05*). Furthermore, under 0.6% mixed salt, the radicle of XHZ is significantly longer than those of the other varieties (*P<0.05*). In conditions of 1.2% Na_2_SO_4_ stress, the radicle of XHZ was significantly longer than that of Shoulu (*P<0.05*). Overall, as the concentration of saline-alkali increases, the radicle length of most *M. ruthenica* resources was inhibited; however, different *M. ruthenica* varieties exhibit specific responses to varying saline-alkali stresses. Similar to plumule length, NaHCO_3_ exhibited the most significant inhibitory effect on the radicle of various *M. ruthenica* species, followed by the effects of mixed salt.

**Table 5:** The radicle length of *Medicago ruthenica* under different saline-alkali stresses.

Concentration	Salt-alkali solutions	Germplasm resources			
		a	b	c	d
0	CK	1.98 ± 0.11ab	1.76 ± 0.23b	2.60 ± 0.22a	2.30 ± 0.32ab
1	A	1.82 ± 0.17a	1.88 ± 0.19a	1.90 ± 0.17a	2.22 ± 0.19a
	B	1.04 ± 0.14b	1.84 ± 0.39ab	2.28 ± 0.28a	1.84 ± 0.24ab
	C	0.34 ± 0.02b	0.38 ± 0.04b	0.74 ± 0.15a	0.74 ± 0.08a
	D	1.14 ± 0.18b	1.60 ± 0.08ab	1.84 ± 0.25a	1.96 ± 0.29a
2	A	1.18 ± 0.06b	1.34 ± 0.05b	1.80 ± 0.23a	1.26 ± 0.14b
	B	1.42 ± 0.06b	2.22 ± 0.28a	1.86 ± 0.24ab	1.84 ± 0.29ab
	C	–	–	–	–
	D	0.46 ± 0.09b	0.92 ± 0.06a	0.44 ± 0.11b	0.32 ± 0.05b
3	A	–	–	–	–
	B	0.90 ± 0.13ab	1.00 ± 0.18a	0.50 ± 0.06b	0.92 ± 0.08ab
	C	–	–	–	–
	D	–	–	–	–

a:YSZ, b:XHZ, c:Shoulu, d:Longzhong 1.

Analysis of the dry weight, fresh weight, and the ratio of dry weight to fresh weight of germinated seedlings indicated that the ratio of dry weight to fresh weight increases with rising saline-alkali concentration. The ratio at a 1.2% concentration is significantly higher than that at a 0.6% concentration (*P< 0.05*), and the ratio at a 0.6% concentration is significantly greater than that at a 0.3% concentration (*P< 0.05*) (**Table 6**). This phenomenon can be attributed to the fact that as the concentration increases, the fresh weight of the germinated seedlings tends to decrease, whereas the dry weight remains stable or may even increase. This suggested that under high saline-alkali stress, the accumulation of dry matter in *M. ruthenica* was relatively enhanced.

**Table 6:** Dry weight, fresh weight and the ratio of dry to fresh weight.

Concentration	Salt-alkali solutions	Germplasm resources											
		a			b			c			d		
		FW	DW	T/R	FW	DW	T/R	FW	DW	T/R	FW	DW	T/R
0	CK	0.102±0.001a	0.009±0.0001a	0.092±0.001g	0.085±0.001a	0.006±0.0001h	0.074±0.001f	0.077±0.001b	0.008±0.0001a	0.099±0.001d	0.120±0.002a	0.010±0.0002b	0.080±0.001f
1	A	0.078±0.001b	0.008±0.0001e	0.098±0.001f	0.078±0.001d	0.008±0.0001f	0.100±0.001d	0.069±0.001d	0.006±0.0001e	0.092±0.002e	0.092±0.002c	0.009±0.0001c	0.101±0.001d
	B	0.068±0.001d	0.007±0.0001f	0.107±0.001e	0.083±0.001b	0.008±0.0001e	0.098±0.001d	0.074±0.001c	0.007±0.0001d	0.092±0.001e	0.088±0.001d	0.009±0.0002d	0.098±0.003e
	C	0.042±0.001g	0.004±0.0001h	0.099±0.003f	0.083±0.001b	0.008±0.0001d	0.100±0.001d	0.062±0.001f	0.006±0.0002f	0.099±0.001d	0.053±0.001g	0.005±0.0001e	0.100±0.001e
	D	0.064±0.001e	0.006±0.0001g	0.090±0.001h	0.080±0.001c	0.007±0.0002g	0.093±0.002e	0.083±0.001a	0.008±0.0001a	0.094±0.002e	0.097±0.002b	0.009±0.0002c	0.096±0.002e
2	A	0.062±0.001f	0.009±0.0001b	0.145±0.001b	0.071±0.001e	0.009±0.0001c	0.123±0.001c	0.066±0.001e	0.007±0.0001c	0.109±0.002c	0.051±0.002g	0.009±0.0001d	0.167±0.004b
	B	0.067±0.001d	0.009±0.0001c	0.132±0.001c	0.078±0.001d	0.009±0.0001a	0.121±0.002c	0.067±0.001e	0.007±0.0001c	0.106±0.002c	0.077±0.001e	0.009±0.0001c	0.121±0.002c
	C	–	–	–	–	–	–	–	–	–	–	–	–
	D	0.070±0.001c	0.009±0.0001c	0.125±0.002d	0.048±0.001g	0.008±0.0001g	0.157±0.004b	0.058±0.001g	0.007±0.0001b	0.127±0.002b	0.042±0.001h	0.005±0.0001e	0.125±0.001c
3	A	–	–	–	–	–	–	–	–	–	–	–	–
	B	0.038±0.001h	0.008±0.0001d	0.208±0.003a	0.052±0.001f	0.009±0.0001b	0.175±0.002a	0.025±0.001h	0.005±0.0001g	0.210±0.007a	0.057±0.001f	0.010±0.0002a	0.183±0.004a
	C	–	–	–	–	–	–	–	–	–	–	–	–
	D	–	–	–	–	–	–	–	–	–	–	–	–

a:YSZ, b:XHZ, c:Shoulu, d:Longzhong 1.

#### 3.1.2 Phenotypic characteristics

**Figure 1:**
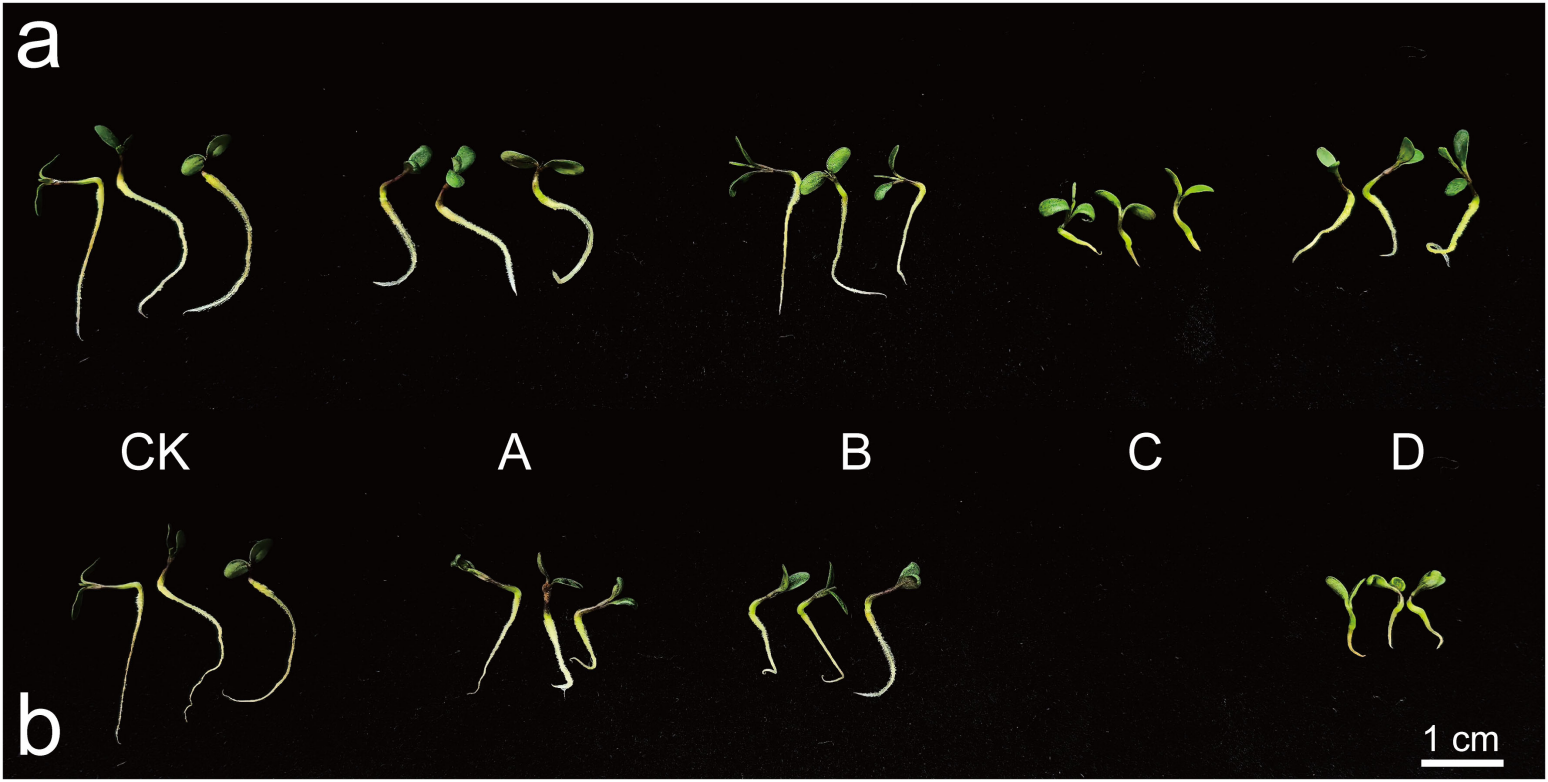
The impact of saline-alkali stress on the phenotype of XHZ’s germlings. In the figures presented, Figure **(a)** illustrates the effects observed at a concentration of 0.3%, while Figure **(b)** depicts the outcomes at a concentration of 0.6%. The experimental conditions include a distilled water control (CK) and various stress treatments: NaCl stress (A), Na_2_SO_4_ stress (B), NaHCO_3_ stress (C), and mixed saline-alkali stress (D).

**Figure 1** illustrateed that at a concentration of 0.3% (**Figure 1a**), the roots of CK were the longest, while NaHCO_3_ exhibitd the strongest inhibitory effect. The bud length is significantly shorter compared to other treatments, and the roots appear curled and shortened, with a marked inhibition of hypocotyl elongation. At a concentration of 0.6% (**Figure 1b**), *M. ruthenica* did not germinate under NaHCO_3_ stress. The root system of the bud seedlings under Na_2_SO_4_ stress was highly curved, and hypocotyl elongation was also inhibited. The inhibitory effects of mixed saline-alkali conditions were particularly pronounced. The bud length remains significantly shorter than that of other treatments, and the development of both the hypocotyl and root system was severely inhibited. These observations provided a clear morphological basis for further investigations into the salt-tolerance mechanisms of *M. ruthenica*.

### 3.2 The impact of saline-alkali stress on the root tip cells of *M. ruthenica*

**Figure 2:**
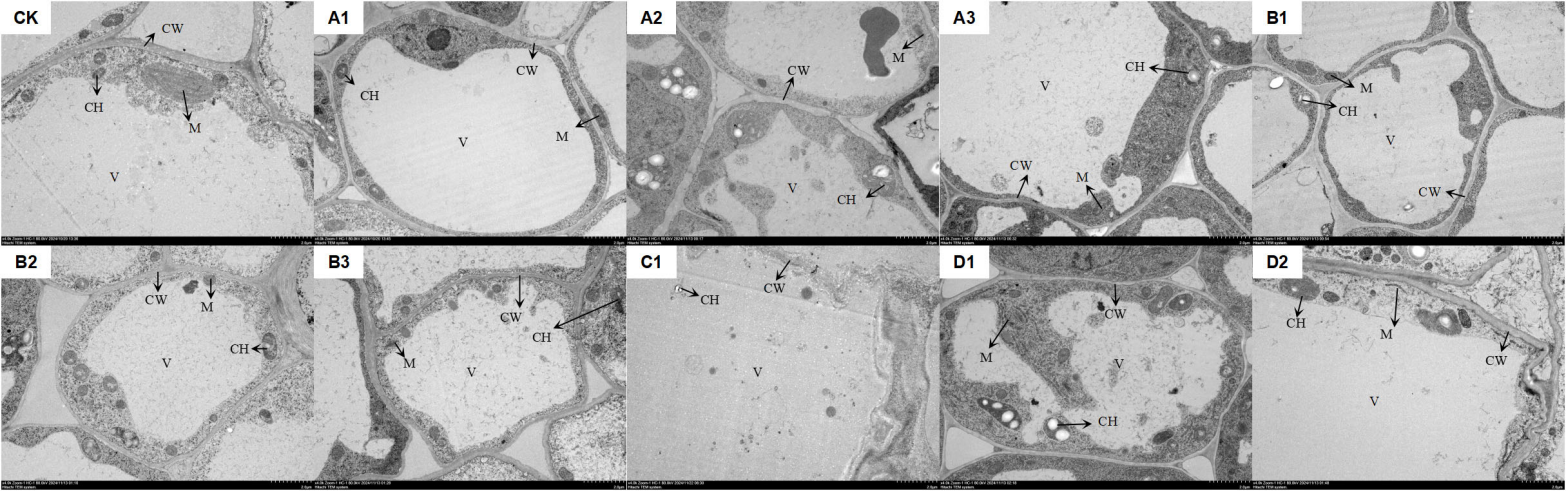
The impact of saline-alkali stress on the ultrastructure of root tip cells of XHZ’s germlings. In the figure, CK represents the distilled water control. **(A1–A3)** correspond to NaCl stress at three concentrations: 0.3%, 0.6%, and 1.2%, respectively. **(B1–B3)** indicate Na_2_SO_4_ stress at the same three concentrations of 0.3%, 0.6%, and 1.2%. **(C1)** represents NaHCO_3_ stress at a concentration of 0.3%. **(D1, D2)** denote mixed saline-alkali stress at concentrations of 0.3% and 0.6%, respectively (the same applies hereinafter). CW, CH, M, and V represent the cell wall, chloroplast, mitochondrion, and vacuole, respectively (the scale is consistently 2μm).

**Figure 2** illustrated that, compared to the control group (CK), various saline-alkali stresses and the same saline-alkali at different concentrations significantly affect the root tip cells of *M. ruthenica*. In the CK group, chloroplasts were elliptical and neatly arranged, while mitochondria are ellipsoidal and positioned adjacent to the chloroplasts. The vacuole boundaries are clear and well-defined. Under NaCl stress (A1-A3), as the concentration of stress increases, chloroplasts expand and enlarge, mitochondria decrease in size, and the vacuole membrane becomes damaged. At a concentration of 1.2%, plasmolysis was observed in the cells. Under Na_2_SO_4_ stress (B1-B3), the cellular changes mirror those seen under NaCl stress; however, plasmolysis occurs at a concentration of 0.6%. When exposed to 0.3% NaHCO_3_ stress, although the seeds can still germinate, the structure of the root tip cells was compromised, lacking complete cellular integrity (C1). In the case of mixed saline-alkali stress (D1, D2), as the concentration increases, chloroplasts swell, the number of thylakoids in the internal matrix diminishes, mitochondria become disordered in arrangement, and some even undergo disintegration. The boundaries of the vacuoles appear blurred and damaged, with plasmolysis occurring at a concentration of 0.6%.

### 3.3 The impact of saline-alkali stress on the metabolites of *M. ruthenica* sprouts

#### 3.3.1 Metabolite qualitative and quantitative analysis

**Figure 3:**
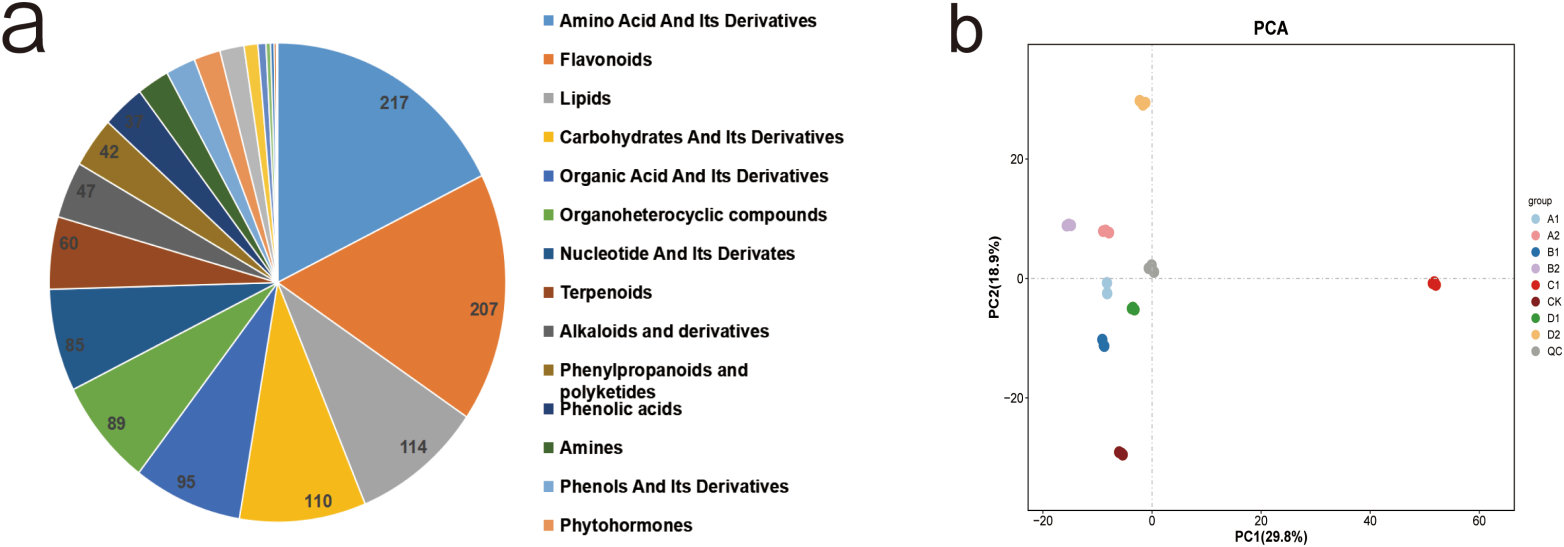
Metabolomic analysis of XHZ seedling germination under different saline-alkali stress conditions. **(a)** Classification of total metabolites.The top three are amino acid and its derivatives, flavonoids, and lipids. **(b)** Principal Component Analysis of Intergroup Metabolic Differences in Seed Germination of XHZ under Various Saline-Alkali Stress Conditions(PCA).

We annotated metabolites based on multiple retrieval databases, detecting a total of 1,230 metabolites across nine groups subjected to different treatments. Among these, there were 777 positive (POS) metabolites and 453 negative (NEG) metabolites. According to the Kyoto Encyclopedia of Genes and Genomes (KEGG) database, the retrieved metabolites were classified into 21 categories. The most abundant category was amino acids and their derivatives, comprising 217 metabolites (18%). This was followed by Flavonoids (207 metabolites, 17%), lipids (114 metabolites, 9%), and sugars and their derivatives (**Figure 3a**). Principal component analysis (PCA) of the samples facilitated visualization of the overall metabolic differences between groups and the degree of variability among samples within the same group. The overall PCA results for all groups and quality control (QC) samples, as depicted in **Figure 3b**, indicate distinct metabolite profiles for each treatment group. The first principal component accounts for 29.8% of the total variability in the dataset, while the second principal component explains 18.9%, demonstrating a complete separation of the samples. Notably, the eight treatment groups were distinctly separated along the first principal component, with the C1 treatment positioned on the right side of PC1 and the other treatments on the left, reflecting varying degrees of stress from different saline-alkali conditions. The stress induced by NaHCO_3_ was more pronounced, while the stress from the other treatments was comparatively weaker. Additionally, the eight treatments exhibit separation along the second principal component; the control and 0.3% concentration stress treatments are located below PC2, whereas the 0.6% concentration stress treatment was positioned above PC2. The arrangement from bottom to top corresponds to increasing stress intensity.

#### 3.3.2 Screening of differentially metabolized substances in different treatments

**Figure 4:**
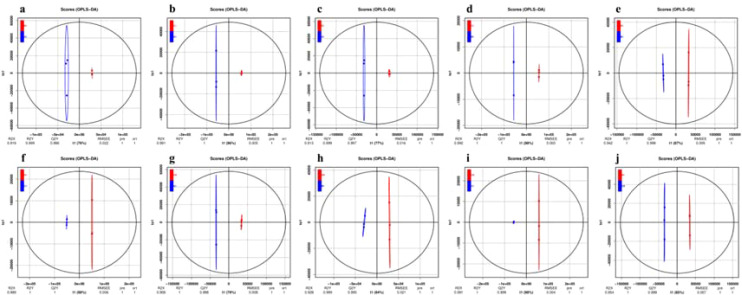
The OPLS-DA score plot of metabolites in germinated seedlings of XHZ under different saline-alkali stress conditions. In this model, R²X(cum) and R²Y(cum) represent the explanatory rates of the constructed model for the X and Y matrices, respectively, while Q² indicates the model's predictive ability. All pairwise comparisons yield high R²X, R²Y, and Q² values, indicating that these analyses are repeatable, reliable, and suitable for screening differential metabolites. **(a)** A1 vs B1. **(b)** A1 vs C1. **(c)** A1 vs D1. **(d)** B1 vs C1. **(e)** B1 vs D1. **(f)** C1 vs D1. **(g)** CK vs A1. **(h)** CK vs B1. **(i)** CK vs C1. **(j)** CK vs D1.

Orthogonal partial least squares discriminant analysis (OPLS-DA) is a multivariate analysis method that relies on multiple independent variable regression modeling. This method is effective for distinguishing differences in pairwise comparisons and enhancing the effectiveness and resolution of Model (Fu et al., 2023). To further illustrated the differences between groups and facilitate identification, various metabolites and their contents were normalized, followed by the application of OPLS-DA models to generate OPLS-DA score maps. In this model, R²X(cum) and R²Y(cum) represented the explanatory rates of the constructed model for the X and Y matrices, respectively, while Q² indicated the model’s predictive ability. The OPLS-DA results (**Figures 4a-j**) revealed that the CK and 0.3% concentration samples under different saline-alkali stress treatments are distinctly separated from each other in pairs. We employed the OPLS-DA model to compare A1 vs B1 (R²X = 0.919, R²Y = 0.999, Q² = 0.996), A1 vs C1 (R²X = 0.991, R²Y = 1, Q² = 1), A1 vs D1 (R²X = 0.913, R²Y = 0.999, Q² = 0.997), B1 vs C1 (R²X = 0.992, R²Y = 1, Q² = 1), B1 vs D1 (R²X = 0.942, R²Y = 1, Q² = 0.999), C1 vs D1 (R²X = 0.989, R²Y = 1, Q² = 1), CK vs A1 (R²X = 0.908, R²Y = 1, Q² = 0.998), CK vs B1 (R²X = 0.928, R²Y = 0.999, Q² = 0.995), CK vs C1 (R²X = 0.991, R²Y = 1, Q² = 0.999), and CK vs D1 (R²X = 0.954, R²Y = 1, Q² = 0.999). All pairwise comparisons yield high R²X, R²Y, and Q² values, indicating that these analyses are repeatable, reliable, and suitable for screening differential metabolites. To prevent overfitting of the supervised model during the modeling process, a permutation test was employed to validate the model’s integrity. As the R² and Q² values of the random model gradually decrease, it suggested that there is no overfitting in the original model (see attachment figures a1-j1), confirming that the separation of metabolites between groups is statistically significant.

**Figure 5:**
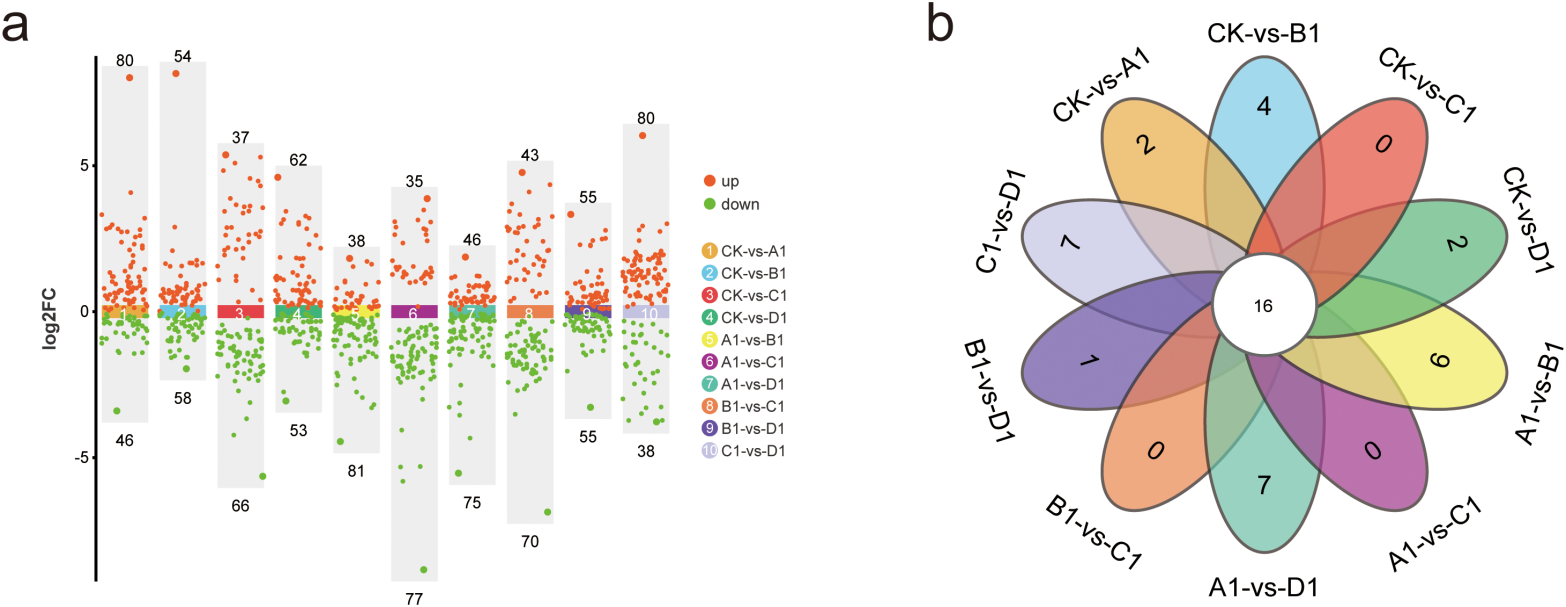
Analysis of metabolite differences in XHZ germinated seedlings under various saline-alkali stress conditions. **(a)** Scatter plot of metabolite differences between different treatments.Scatter plots are primarily utilized to illustrate the screened differential metabolites. In this study, the screening of differential metabolites is conducted based on the criteria of p-value 1. **(b)** Venn diagram of metabolite differences between different treatments.

Scatter plots were primarily utilized to illustrate the screened differential metabolites. In this study, the screening of differential metabolites was conducted based on the criteria of p-value 1. The results of the screening are depicted in scatter plots (**Figure 5a**). A total of 126 significantly different metabolites were identified between CK and A1 (80 up-regulated and 46 down-regulated), 112 between CK and B1 (54 up-regulated and 58 down-regulated), 103 between CK and C1 (37 up-regulated and 66 down-regulated), 115 between CK and D1 (62 up-regulated and 53 down-regulated), 119 between A1 and B1 (38 up-regulated and 81 down-regulated), 112 between A1 and C1 (35 up-regulated and 77 down-regulated), 121 between A1 and D1 (46 up-regulated and 75 down-regulated), 113 between B1 and C1 (43 up-regulated and 70 down-regulated), 110 between B1 and D1 (55 up-regulated and 55 down-regulated), and 118 between C1 and D1 (80 up-regulated and 38 down-regulated). We intuitively analyzed the similarities and differences among the various treatments and generated a Venn diagram (**Figure 5b**). Notably, no specific differential metabolites were identified between C1 and A1 or B1, while specific differential metabolites were observed between the other treatment comparisons.

In addition, 16 common differential metabolites were identified, which may serve as potential biomarkers for distinguishing between different saline-alkali stresses. The detailed information regarding these 16 common differential metabolites, after normalization, was presented in **Table 7**. Notably, among these 16 differential metabolites, 12 are amino acids and their derivatives, 3 are organic acids and their derivatives, and 1 is a flavonoid. Among the ten most common differential metabolites, the contents of isoleucine, L-leucine, 4-aminobutyric acid, L-arginine, histidine, glutamine, L-lysine, and apigenin 7-*O*-beta-D-glucuronide in broad bean sprouts subjected to alkali stress are relatively the lowest, followed by those treated with a mixed salt of saline and alkali. In contrast, the contents of cycloleucine and citric acid in the alkali treatment were comparatively higher. Therefore, cycloleucine and citric acid may emerge as key metabolites for distinguishing saline-alkali stress in future studies.

**Table 7:** 16 kinds of common differential metabolites.

Sequences	Compounds	Class	CK(log10FC)	A1(log10FC)	B1(log10FC)	C1(log10FC)	D1(log10FC)
1	isoleucine	Amino Acid And Its Derivatives	8.55 ± 0.005	8.77 ± 0.006	8.74 ± 0.003	8.31 ± 0.005	8.64 ± 0.003
2	L-Leucine	Amino Acid And Its Derivatives	8.33 ± 0.006	8.61 ± 0.008	8.55 ± 0.003	8.08 ± 0.006	8.44 ± 0.004
3	4-Aminobutyric acid	Amino Acid And Its Derivatives	7.39 ± 0.008	7.52 ± 0.005	7.57 ± 0.010	6.94 ± 0.009	7.67 ± 0.003
4	L-arginineL	Amino Acid And Its Derivatives	8.60 ± 0.006	8.48 ± 0.003	8.57 ± 0.002	8.22 ± 0.001	8.41 ± 0.006
5	Histidine	Amino Acid And Its Derivatives	8.51 ± 0.004	8.37 ± 0.003	8.42 ± 0.002	7.96 ± 0.013	8.32 ± 0.003
6	Glutamine	Amino Acid And Its Derivatives	8.53 ± 0.003	8.44 ± 0.004	8.48 ± 0.002	7.71 ± 0.009	8.24 ± 0.007
7	Cycloleucine	Amino Acid And Its Derivatives	7.78 ± 0.002	7.84 ± 0.003	7.72 ± 0.004	8.15 ± 0.003	7.91 ± 0.018
8	L-lysine	Amino Acid And Its Derivatives	8.47 ± 0.004	8.43 ± 0.004	8.45 ± 0.002	7.76 ± 0.004	8.26 ± 0.005
9	Citric acid	Organic Acid And Its Derivatives	7.20 ± 0.003	7.89 ± 0.004	7.40 ± 0.004	8.28 ± 0.007	8.16 ± 0.007
10	Apigenin7-O-beta-D-glucuronide	Flavonoids	7.53 ± 0.006	7.26 ± 0.013	7.36 ± 0.008	6.89 ± 0.003	7.48 ± 0.013
11	D-Histidine	Amino Acid And Its Derivatives	7.52 ± 0.010	7.30 ± 0.003	7.37 ± 0.002	6.85 ± 0.002	7.21 ± 0.002
12	Aspartic Acid	Amino Acid And Its Derivatives	7.59 ± 0.005	7.79 ± 0.006	7.72 ± 0.005	8.25 ± 0.005	7.93 ± 0.004
13	D-threo-Isocitric acid	Organic Acid And Its Derivatives	6.82 ± 0.006	7.47 ± 0.015	7.04 ± 0.005	7.86 ± 0.002	7.73 ± 0.007
14	L-Histidine	Amino Acid And Its Derivatives	7.86 ± 0.008	7.64 ± 0.004	7.67 ± 0.012	7.17 ± 0.007	7.55 ± 0.004
15	Isocitrate	Organic Acid And Its Derivatives	7.31 ± 0.019	7.99 ± 0.009	7.52 ± 0.003	8.39 ± 0.005	8.26 ± 0.004
16	Iminodiacetic acid	Amino Acid And Its Derivatives	7.57 ± 0.014	7.76 ± 0.007	7.72 ± 0.002	8.22 ± 0.006	7.90 ± 0.006

#### 3.3.3 KEGG pathway annotation of differential metabolites

**Figure 6:**
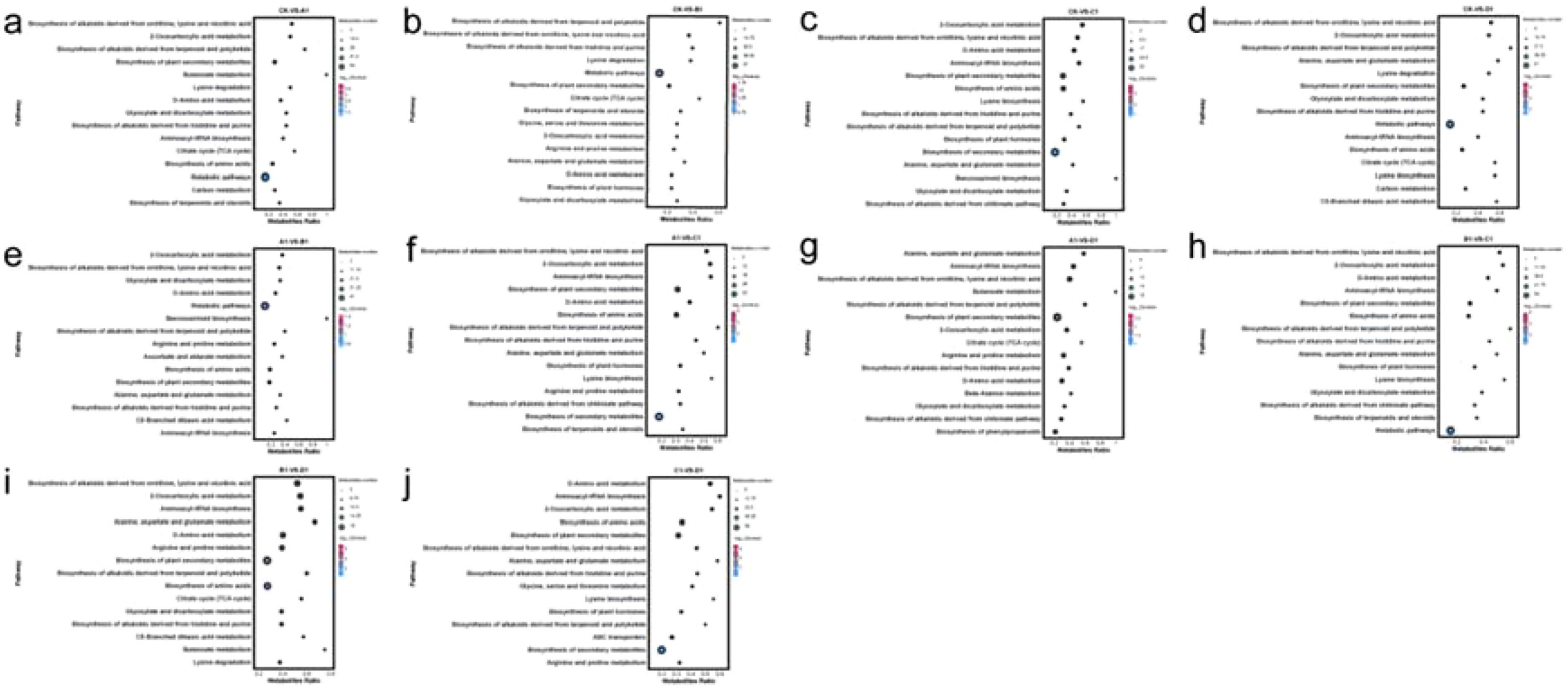
KEGG enrichment of metabolites in XHZ germinated seedlings under different saline-alkali stress conditions. The x-axis represents the enrichment score, while the y-axis displays the information pertaining to the top 20 pathways. Pathways represented by larger bubbles indicate a greater number of differential metabolites. Additionally, the color of the bubbles transitions from blue to red, signifying a decrease in the enrichment p-value, thus indicating greater statistical significance. Panels differ in pathway prominence and significance levels. **(a)** CK-VS-A1, **(b)** CK-VS-B1, **(c)** CK-VS-C1, **(d)** CK-VS-D1, **(e)** A1-VS-B1, **(f)** A1-VS-C1, **(g)** A1-VS-D1, **(h)** B1-VS-C1, **(i)** B1-VS-D1, **(j)** C1-VS-D1.

The KEGG database (https://www.genome.jp/kegg/) serves as a crucial resource for understanding metabolic pathways. Through pathway enrichment analysis of differential metabolites, we gained insights into the mechanisms underlying changes in metabolic pathways across different samples. In this study, we identified and classified the differential metabolites in each group according to their respective pathways. Furthermore, the differential metabolites between the groups A1 and B1, A1 and C1, A1 and D1, B1 and C1, B1 and D1, C1 and D1, CK and A1, CK and B1, CK and C1, and CK and D1 correspond to 73, 80, 76, 78, 75, 81, 83, 76, 76, and 76 pathways, respectively. The primary pathways were illustrated in **Figures 6a-j**. Notably, among the ten comparison groups, seven groups exhibit the most significant enrichment in the biosynthesis of alkaloids derived from ornithine, lysine, and nicotinic acid. The remaining three groups with the most significant enrichment pertain to aminoacyl-tRNA biosynthesis, D-amino acid metabolism, and 2-oxocarboxylic acid metabolism. The biosynthesis of alkaloids derived from ornithine, lysine, and nicotinic acid demonstrates particularly notable enrichment.

The differential metabolic pathways between the C1 and D1 comparison groups exhibit significant differences when contrasted with those of other comparison groups. Notably, D-Amino acid metabolism and Aminoacyl-tRNA biosynthesis were more prominently enriched in the C1 and D1 comparison group. In contrast, the pathways that showed that greater enrichment in the other comparison groups primarily include the biosynthesis of alkaloids derived from ornithine, lysine, and nicotinic acid, as well as 2-oxocarboxylic acid metabolism. This disparity may be attributed to the fact that the addition of NaCl and Na_2_SO_4_ in the D1 treatment modifies its metabolic pathways in comparison to the C1 treatment.

### 3.4 Analysis of the correlation between germination indicators and metabolic indicators

The Mantel test and Pearson correlation analysis were utilized to examine the relationship between the germination indicators of XHZ (GE, GR, RL, BL, FW, DW, T/R) and key metabolites. From the perspective of metabolites, isoleucine and L-Leucine exhibited a highly significant positive correlation (P<0.0001), as well as a significant positive correlation with 4-Aminobutyric acid (P<0.01). L-arginine demonstrated significant positive correlations with histidine, glutamine, and D-histidine (*P<0.01*), while showing significant negative correlations with cycloleucine (*P<0.01*), aspartic acid (*P<0.001*), and iminodiacetic acid (*P<0.01*). Additionally, histidine and glutamine (*P<0.01*), L-lysine (*P<0.01*), D-histidine (*P<0.001*), and L-histidine (*P<0.01*) were found to be extremely significantly positively correlated, whereas they exhibited extremely significant negative correlations with aspartic acid and iminodiacetic acid (*P<0.01*). Furthermore, glutamine and L-lysine (*P<0.001*), D-histidine (*P<0.01*), and L-histidine (*P<0.01*) displayed extremely significant positive correlations, alongside extremely significant negative correlations with cycloleucine, aspartic acid, and iminodiacetic acid (*P<0.01*). Cycloleucine and L-lysine exhibit extremely significant positive correlations (*P<0.001*), as well as extremely significant negative correlations with Cycloleucine, Aspartic Acid, and Iminodiacetic acid (*P<0.01*). Furthermore, Cycloleucine shows an extremely significant negative correlation with L-lysine (*P<0.01*). Additionally, L-lysine and D-Histidine demonstrated a highly significant positive correlation (*P<0.01*) and a highly significant negative correlation with Aspartic Acid and Iminodiacetic acid (*P<0.01*). Citric acid was also found to have a highly significant positive correlation with D-threo-Isocitric acid and Isocitrate (*P<0.0001*). D-Histidine and L-Histidine were positively correlated with high significance (*P<0.001*) and negatively correlated with Aspartic Acid and Iminodiacetic acid (*P<0.001*). Moreover, Aspartic Acid and Iminodiacetic acid exhibited a highly significant positive correlation (*P<0.0001*) and a highly significant negative correlation with L-Histidine (*P<0.001*). Lastly, D-threo-Isocitric acid and Isocitrate also presented a highly significant positive correlation (*P<0.0001*), while L-Histidine shows a highly significant negative correlation with Iminodiacetic acid (*P<0.001*).

**Figure 7:**
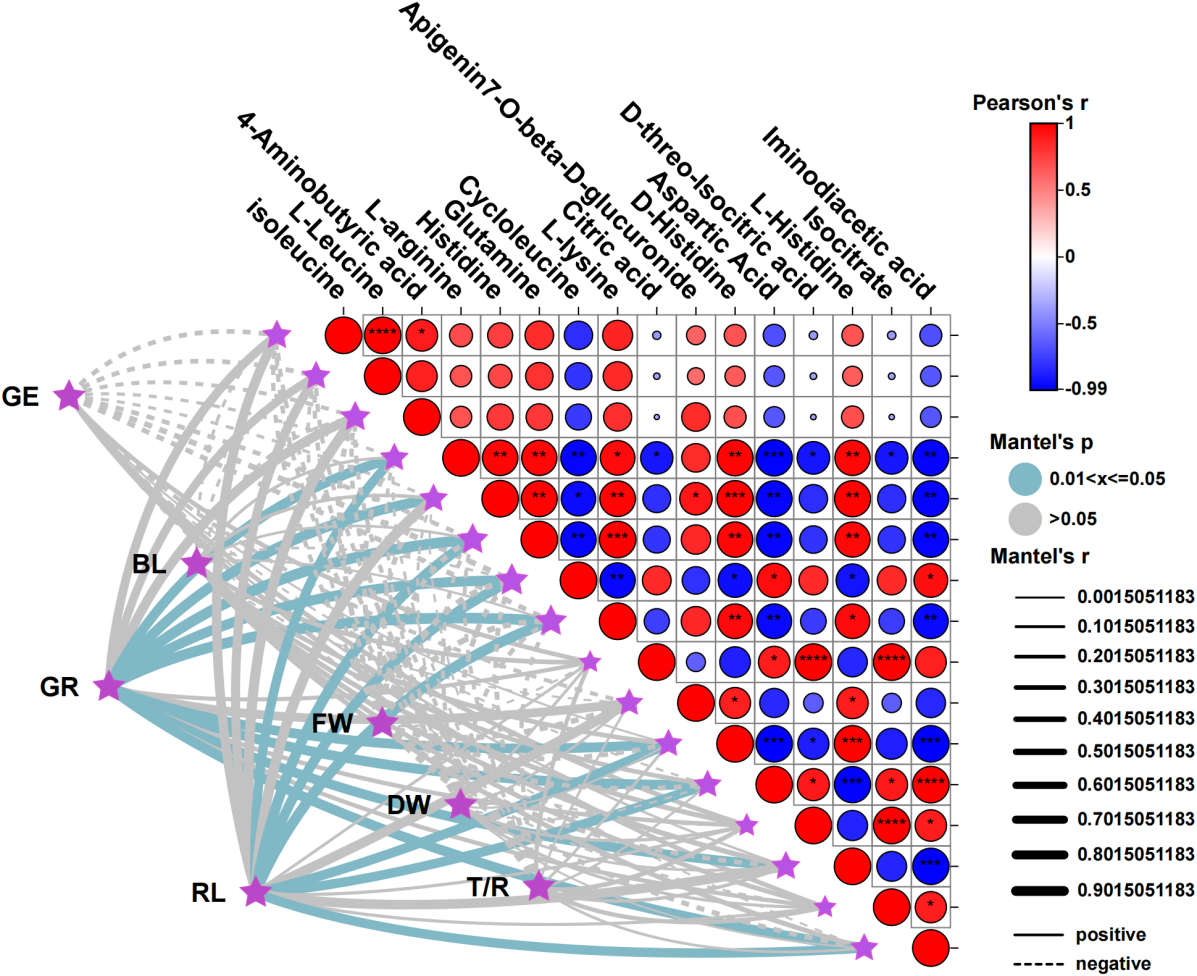
Interactive Mantel test-related heat map analysis of germination indicators and key metabolites. The vertical coordinate represents key metabolites, and the horizontal coordinate represents the germination index. *P <0.05, **P < 0.01, ***P < 0.001. GE, germination energy; GR, germination rate; BL, bud length; RL, root length; FW, fresh weight; DW, dry weight; T/R, dry-to-fresh weight ratio.

In terms of germination indicators and metabolites, GR was significantly positively correlated with nine metabolites, including L-arginine, histidine, glutamine, cycloleucine, and L-lysine. RL was significantly positively correlated with seven metabolites, excluding histidine and L-histidine from the aforementioned metabolites. Notably, all nine metabolites belong to amino acids and their derivatives, indicating that amino acids play a crucial role in the germination rate and root length of *M. ruthenica* seeds during the germination process under saline-alkali stress (**Figure 7**).

## 4 Discussions

### 4.1 The impact of saline-alkali stress on the seed germination of *M. ruthenica*

The response of seed germination to environmental changes, such as soil salinity, is a crucial factor influencing the colonization ability of plants (Gorai and Neffati, 2007). The successful establishment of plants largely depends on the success of germination, particularly under saline-alkali conditions (Li et al., 2010a). It is widely accepted that osmotic and ionic effects are the primary factors inhibiting seed germination under salt stress (Debez et al., 2004). This study found that varying concentrations of saline-alkali stress significantly impact the germination characteristics of *M. ruthenica* seeds. Notably, low-concentration NaCl stress did not significantly inhibit the germination of *M. ruthenica* seeds and even promoted germination to some extent, which aligns with previous research findings (Hasegawa et al., 2000). The seed germination rate of halophytes such as *Haloxylon ammodendron* (Zhumabekova et al., 2020), *Halostachys caspica* (Assareh et al., 2010), and *Centaurea ragusina* (Radić et al., 2013) remains relatively high under low concentrations of neutral salts (Na_2_SO_4_ or NaCl). In contrast, under 200 mmol/L NaCl stress, the seed germination of the annual halophyte *Cakile maritima* was severely inhibited and delayed, resulting in reduced seedling length (Debez et al., 2018). However, as the concentration of saline-alkali increases, both the germination potential and germination rate of *M. ruthenica* seeds decrease significantly. Particularly under high concentrations of NaHCO_3_ and mixed salt, the seeds nearly completely lose their germination ability. This further indicated that alkali-salt stress was more detrimental to plants than neutral salt stress (Yang et al., 2008; Bernstein et al., 2010; Pang et al., 2016; Lin et al., 2017).

There are significant differences in the germination characteristics of *M. ruthenica* germplasm resources from various sources when exposed to saline-alkali stress. The germplasm XHZ exhibits a higher germination potential and rate under low to medium concentrations of saline-alkali stress, indicating robust tolerance to saline-alkali conditions. In contrast, the germination abilities of YSZ and Longzhong 1 markedly decline under high concentrations of saline-alkali stress, suggesting a lower tolerance to these conditions. This finding was consistent with the results reported by Li et al (Wu et al., 2022a), which imply that XHZ may have accumulated a greater number of saline-alkali tolerance genes throughout the long-term domestication process, thereby demonstrating enhanced adaptability.

Saline-alkali stress not only affects seed germination but also significantly impacts the growth of sprout seedlings. This study found that as the concentration of saline-alkali increases, the lengths of the plumule and radicle of *M. ruthenica* sprout seedlings decrease markedly. Notably, under high concentrations of NaHCO_3_ and composite saline-alkali stress, the growth of sprout seedlings was completely inhibited. This observation aligns with the findings of Shi and Sheng (Shi and Sheng, 2005), indicating that the inhibitory effect of saline-alkali stress, particularly at high pH levels, on plant growth is pronounced. Furthermore, it was important to highlight that the growth performance of *M. ruthenica* germplasm resources from various sources exhibits significant differences under saline-alkali stress. For instance, XHZ demonstrated longer plumules and radicles under low to medium concentrations of saline-alkali stress, while it shows more pronounced advantages in root length at high concentrations, suggesting a strong tolerance to saline-alkali conditions. This observation was consistent with previous research indicating that plants can adapt to saline-alkali stress by increasing their root-to-shoot ratio and specific root length (Liu et al., 2015; Wang et al., 2018).

### 4.2 The impact of saline-alkali stress on the ultrastructure of root tip cells of *M. ruthenica*

The speed of seed germination was closely linked to the rate of water absorption. Salinity impedes water absorption, hinders the repair of cell membranes during this process, and exacerbates damage to the membrane structure, leading to the exudation of solutes from the seeds (Yang et al., 2007). As salinity levels increase, the degree of damage to the seeds intensifies, ultimately affecting germination (Yang et al., 2008; Bernstein et al., 2010; Li et al., 2019). Moreover, saline-alkali stress can disrupt plant cell structure, destabilize osmotic pressure, and interfere with nutrient absorption (Gong et al., 2016; Ruiz et al., 2016). This study utilized transmission electron microscopy to observe that saline-alkali stress significantly impacts the ultrastructure of root tip cells in *M. ruthenica*. With increasing saline-alkali concentration, notable changes occur in the structure of chloroplasts and mitochondria within root tip cells. Under high concentrations of NaCl and Na_2_SO_4_, plasmolysis is observed in the cells. This phenomenon aligns with findings from previous studies, where higher osmotic pressure leads to increased plasmolysis of the cytoplasm, inhibiting seed expansion. Additionally, the toxic effects of high salt ion concentrations can inhibit enzyme activity and disrupt intracellular metabolic processes, resulting in a decline in all germination indicators (Ibrahim, 2016; Debez et al., 2018).

### 4.3 The impact of saline-alkali stress on the metabolites of *M. ruthenica*

Numerous studies have demonstrated significant differences between mixed saline-alkali stress and individual saline or alkali stress, revealing a more complex scenario than that presented by single stressors (Li et al., 2010b; Wang et al., 2022). A key characteristic of mixed saline-alkali stress is the mutual enhancement of salt and alkali stress on plants. In natural environments, high salinity and elevated pH values often coexist, and their synergistic effects may exert a greater influence on plant growth and development than either stress alone. Furthermore, the impacts of salt stress and alkali stress on plants exhibit notable differences. Metabolomics analysis in this study indicates that, under saline-alkali stress, significant alterations have occurred in key metabolic pathways, including amino acid metabolism, flavonoid metabolism, and lipid metabolism in the germinated seedlings of *M. ruthenica*. Notably, the accumulation of amino acids and their derivatives has increased significantly, aligning with the findings of Ashraf (Ashraf and Foolad, 2007). In higher plants, amino acids accumulated in response to various stresses and play multiple roles in plant growth (Less and Galili, 2008). The accumulation of amino acids was specifically a response to salt stress (Mutwakil et al., 2017).

Under salt stress and the combined stress of salt and alkali, the accumulation of isoleucine, L-leucine, 4-aminobutyric acid, citric acid, and other osmotic adjustment substances in the bud seedlings of *M. ruthenica* increased significantly. These substances play crucial roles in maintaining cellular osmotic balance and enhancing antioxidant defense (Wu et al., 2013). Furthermore, saline-alkali stress prompts *M. ruthenica* bud seedlings to produce a substantial amount of secondary metabolites, including flavonoids and polyphenols, which are important for antioxidant activity and signal transduction (Nakabayashi et al., 2014). Notably, this study identified that cycloleucine and citric acid as potential key metabolites for differentiating salt stress from alkali stress in future research. Previous studies have shown that citric acid can enhance plant height, leaf water content, and the activity of antioxidant enzymes such as peroxidase in *Festuca elata*. It also promotes chlorophyll synthesis and mitigates salt damage in tall fescue (Tahjib-Ul-Arif et al., 2021). Additionally, research on cycloleucine indicates that it significantly inhibits plant growth (Niu et al., 2023). KEGG pathway annotation revealed distinct differences in the significantly enriched pathways associated with salt stress, alkali stress, and mixed saline-alkali stress. The primary enriched pathway for alkali stress is 2-oxocarboxylic acid metabolism, while the main enriched pathways for salt stress and mixed saline-alkali stress involve the biosynthesis of alkaloids derived from ornithine, lysine, and nicotinic acid.

Relevance analysis indicated that amino acids and their derivatives play a crucial role in the seed germination and seedling growth of *M. ruthenica*. Notably, amino acids such as L-arginine, histidine, and glutamine exhibit a significan positive correlation with both germination rate and root length. This correlation can be attributed to the specific functions of these amino acids. Nitric oxide (NO), which was synthesized from L-arginine, has been shown to alleviate seed dormancy, promote germination, and enhance the germination rate. Furthermore, NO stimulate the formation of adventitious and lateral roots by regulating auxin, thereby facilitating root expansion. In Additionally, both histidine and glutamine serve as osmotic adjustment substances, regulating cellular osmotic pressure, aiding in water retention, and maintaining cell turgor, which ultimately enhances plant stress resistance. These findings align with the research conducted by Fiehn (Fiehn, 2002), underscoring the significant role of amino acid metabolism in plant stress responses.

Future research can further investigate the molecular regulatory mechanisms of *M. ruthenica* under saline-alkali stress, particularly focusing on the gene expression regulation network of key metabolic pathways. This exploration aimed to provide theoretical support for the molecular breeding of saline-alkali-tolerant forage. Furthermore, by integrating multi-omics technologies such as transcriptomics and proteomics, a more comprehensive understanding of the adaptation mechanisms of *M. ruthenica* under saline-alkali stress can be achieved, thereby offering a scientific basis for the ecological restoration and sustainable utilization of saline-alkali land.

## 5 Conclusion

This research systematically investigated the seed germination characteristics and metabolic response mechanisms of *M. ruthenica* under saline-alkali stress. The findings indicated that saline-alkali stress significantly impacts the germination and seedling growth of *M. ruthenica* seeds. Notably, high concentrations of NaHCO_3_ and composite saline-alkali stress exert a pronounced inhibitory effect on seed germination and seedling development. There were substantial differences in the germination characteristics of *M. ruthenica* germplasm resources from various sources when subjected to saline-alkali stress. Specifically, XHZ exhibited strong saline-alkali tolerance, suggesting that it may have accumulated a greater number of saline-alkali tolerance genes throughout the long-term domestication process. Furthermore, metabolomics analysis revealed significant alterations in the metabolite profile of *M. ruthenica* sprouts under saline-alkali stress. In particular, there was a notable increase in the accumulation of amino acids and their derivatives. These metabolites were crucial for maintaining cellular osmotic balance and antioxidant defense. Correlation analysis demonstrated a close relationship between amino acids and their derivatives and the seed germination and sprout growth of *M. ruthenica*. Notably, amino acids such as L-arginine, histidine, and glutamine exhibited significant positive correlations with the germination rate and root length, indicating their vital role in *M. ruthenica*’s response to saline-alkali stress.

## Data Availability

The original contributions presented in the study are included in the article/**Supplementary Material**. Further inquiries can be directed to the corresponding authors.
