# Supplementary figures and images for: Analysis of germination characteristics and metabolome of Medicago ruthenica in response to saline-alkali stress
Source: Front Plant Sci. 2025 Jul 1;16:1592555. doi: 10.3389/fpls.2025.1592555 (PMC12259608; doi:10.3389/fpls.2025.1592555)

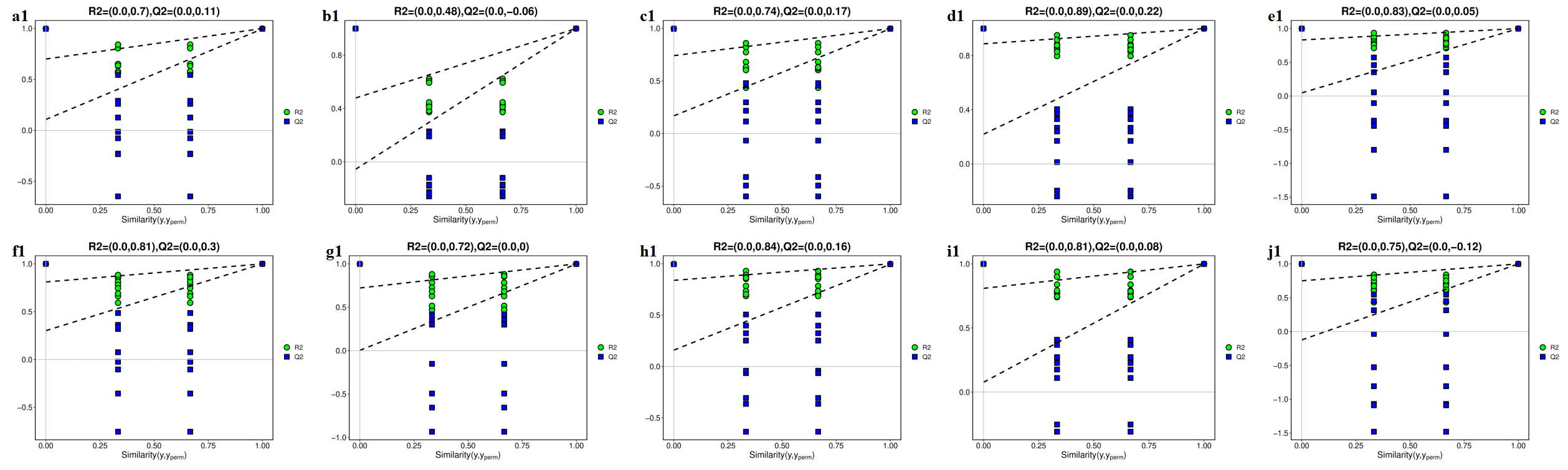

Supplement: Supplementary file 1 [file Image1.png]
